# Revisiting the Social Frailty Index: A Replication With Implications for an Emerging Construct

**DOI:** 10.1093/geroni/igaf122.4186

**Published:** 2025-12-31

**Authors:** Charles Collinge, Monica Luciana

**Affiliations:** University of Minnesota, Minneapolis, Minnesota, United States; University of Minnesota, Minneapolis, Minnesota, United States

## Abstract

Frailty is a manifestation of the aging process associated with adverse outcomes. Methods to quantify it typically focus on physical decline. However, formulations that include social behavior are gaining interest. Recently, Shah and colleagues (2023) developed a 10-item Social Frailty Index (SFI-10) in data from the Health and Retirement Study (HRS) that used 8 social items plus age and sex to successfully predict mortality. This study evaluates the construct and criterion validity of the SFI-10. Utilizing Shah (2023)’s HRS cohorts (n = 8264, ages 66 to 101), and a sample from the Midlife in the United States (MIDUS) study (n = 5619, ages 40 to 75), we constructed the SFI-10 in both samples alongside an 8-item index (SFI-8) that reflects only social behavior. Logistic regression was used to predict mortality from the SFI-10, SFI-8, and models that included only age and sex from midlife to old age. Within MIDUS, we also evaluated the SFI-8, SFI-10, and age against criteria relevant to social frailty. Findings indicate that the SFI-10 relies heavily on age, with marginal contribution from its social items, to predict mortality in the HRS. Although social items had heightened importance for mortality prediction in MIDUS, the reliance on age persisted. While associations were observed between the SFI-10 and external criteria relevant for social frailty, the SFI-8 exhibited even stronger associations with these criteria. These findings raise questions about how social frailty should be quantified. A measure that exclusively indexes social behavior may best reflect the social frailty construct.

